# Bacteriological diagnosis of childhood TB: a prospective observational study

**DOI:** 10.1038/s41598-017-11969-5

**Published:** 2017-09-18

**Authors:** Andrew J. Brent, Daisy Mugo, Robert Musyimi, Agnes Mutiso, Susan C. Morpeth, Michael Levin, J. Anthony G. Scott

**Affiliations:** 1 KEMRI-Wellcome Trust Research Programme, Kilifi, Kenya; 2 University of Oxford, Oxford, UK; 3 Imperial College London, London, UK; 4 Oxford University Hospitals NHS Foundation Trust, Oxford, UK; 5 London School of Hygiene & Tropical Medicine, London, UK

## Abstract

Childhood TB diagnosis is challenging. Studies in adults suggest Microscopic Observation Drug Susceptibility (MODS) culture or the Xpert MTB/RIF assay might be used to expand bacteriological diagnosis. However data from children are more limited. We prospectively compared MODS and Xpert MTB/RIF with standard microscopy and culture using the BD MGIT 960 system among 1442 Kenyan children with suspected TB. 97 specimens from 54 children were TB culture-positive: 91 (94%) by MGIT and 74 (76%) by MODS (p = 0.002). 72 (74%) culture-positive and 7 culture-negative specimens were Xpert MTB/RIF positive. Xpert MTB/RIF specificity was 100% (99.7–100%) among 1164 specimens from 892 children in whom TB was excluded, strongly suggesting all Xpert MTB/RIF positives are true positives. The sensitivity of MGIT, MODS and Xpert MTB/RIF was 88%, 71% and 76%, respectively, among all 104 true positive (culture and/or Xpert MTB/RIF positive) specimens. MGIT, MODS and Xpert MTB/RIF on the initial specimen identified 40/51 (78%), 33/51 (65%) and 33/51 (65%) culture-confirmed pulmonary TB cases, respectively; Xpert MTB/RIF detected 5 additional culture-negative cases. The high sensitivity and very high specificity of the Xpert MTB/RIF assay supports its inclusion in the reference standard for bacteriological diagnosis of childhood TB in research and clinical practice.

## Introduction

Definitive diagnosis of tuberculosis (TB) rests on detection of *M*. *tuberculosis* from clinical specimens, but is difficult in children. While sputum smear microscopy underpins the WHO DOTS and Stop TB strategies for TB diagnosis in adults^1^, obtaining sputum is more difficult in children, and the yield of microscopy alone is very poor due to small numbers of bacilli in clinical specimens. Bacteriological diagnosis therefore depends on mycobacterial culture^2^.

Strengthening laboratory capacity is a key component of the Stop TB Strategy and Global Plan to Stop TB^1,3^. Commercial liquid culture methods like the BACTEC Mycobacteria Growth Indicator Tube (MGIT) system (BD Diagnostics, Sparks, MD, USA) offer higher sensitivity and more rapid results than traditional solid media. However, barriers to their uptake include cost, a lack of technically trained personnel, and the need for biosafety level 3 facilities^3^.

Microcolony culture techniques such as the Microscopic Observation Drug Susceptibility (MODS) assay have several potential advantages over conventional culture methods in low resource settings, including high sensitivity; more rapid results that include drug susceptibility testing (DST); lower cost; and less stringent biosafety requirements making it applicable in biosafety level 2 facilities^4^. However only limited data exist on their use in children^5–7^, and no studies have compared MODS with MGIT for diagnosis of TB in young children; nor evaluated MODS yield from induced sputum samples, despite good evidence and international recommendations supporting sputum induction for childhood TB diagnosis^2,8^.

The Xpert MTB/RIF real-time PCR assay (Cepheid, Sunnyvale, CA, USA) offers a rapid and operationally simpler alternative to culture for detection of *M*. *tuberculosis* and rifampicin resistance^9,10^. A recent meta-analysis suggests modest sensitivity and high specificity for paediatric TB diagnosis^11^, and the latest WHO childhood TB guidelines (2014) recommend Xpert MTB/RIF as an alternative to conventional microscopy and culture - but acknowledge the “very low quality of evidence”^2^.

We compared microscopy, MGIT and MODS culture, and the Xpert MTB/RIF assay for diagnosis of childhood TB in Kenya.

### Participants

We prospectively assessed each diagnostic method among children presenting to Coast Provincial General Hospital and Kilifi County Hospital in Coast Province, Kenya. Between July 2010 and December 2011 children aged <15 years were investigated for TB if one or more of the following features of suspected TB were present and they were not already on TB treatment: unexplained persistent cough for >2 weeks; pneumonia not responding to first line antibiotics; unexplained fever for >2 weeks; unexplained progressive weight loss or failure to thrive for >4 weeks; a history of close contact with a suspected or confirmed case of pulmonary TB; or clinical suspicion of TB for any other reason.

### Clinical procedures

Each child underwent a structured history and examination, chest radiography and tuberculin skin testing (TST) according to WHO guidelines^2^. Severe malnutrition was defined as weight for age z-score of <−3 or the presence of nutritional oedema^12^. Provider initiated testing and counseling for HIV was performed according to Kenyan national guidelines, which recommend testing for all inpatients, and for all patients investigated for TB, on an opt-out basis.

Children who were able to expectorate provided up to three spontaneous sputum samples. Sputum induction was performed on the remainder. If sputum induction was contraindicated (e.g. due to severe respiratory distress), gastric aspiration was performed. Sputum induction and gastric aspiration were performed according to international recommendations^2^. Further investigations including extra-pulmonary or repeat sputum sampling were performed at the discretion of the clinical team caring for the patient.

**Figure 1:**
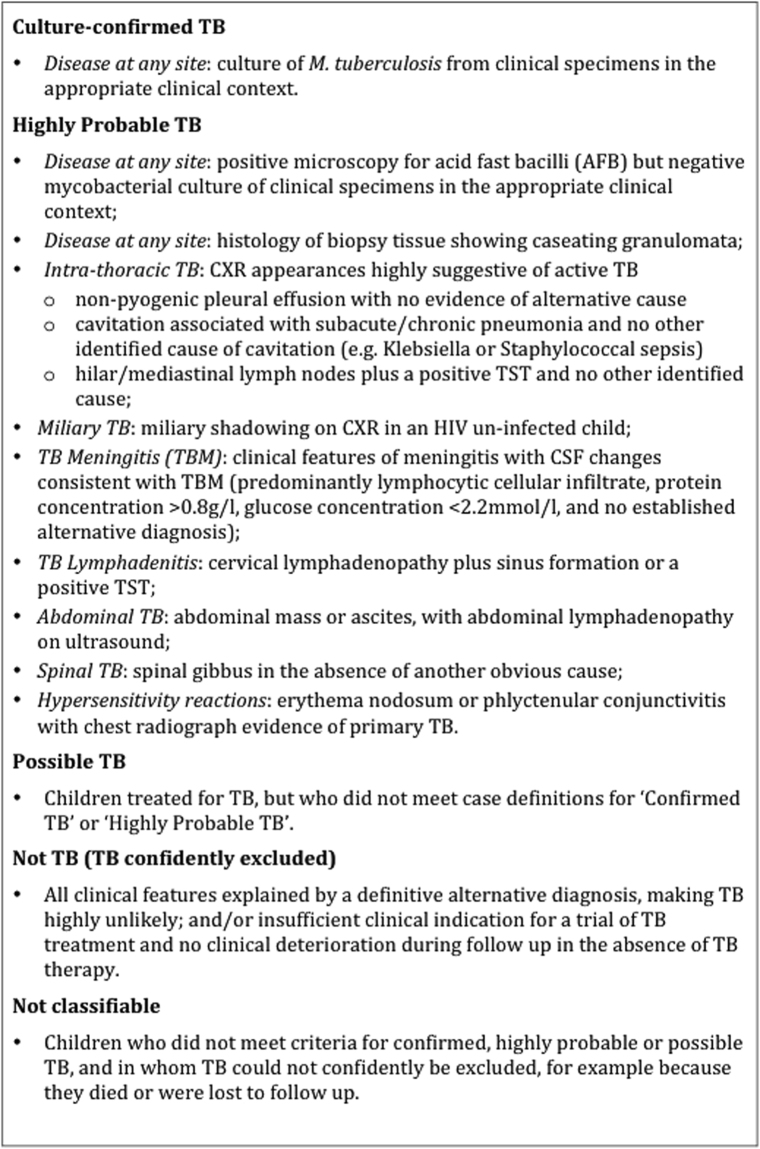
Childhood TB Case Definitions.

We classified children as Confirmed TB, Highly Probable TB, Possible TB or Not TB according to their clinical, radiological and microbiological findings. Categories were defined *a priori* and based closely on published definitions (Fig. 1)^13,14^. Treatment protocols followed Kenyan national guidelines. Children treated for TB were followed up until completion of treatment at 6 months. Other children were also followed up for 6 months, or until TB could be confidently excluded. Final diagnostic assignments were revised in the light of follow up data.

### Laboratory procedures

Specimens were transported to the laboratory at 2–8 °C and processed the same day. Sputum specimens were decontaminated using the modified Petroff’s method and 4% sodium hydroxide^15^; centrifuged; and the sediment re-suspended in 2 ml culture broth containing Middlebrook 7H9 medium, oxalic acid, albumin, dextrose, and catalase (OADC, Becton Dickinson), and polymyxin, amphotericin B, nalidixic acid, trimethoprim, and azlocillin (PANTA, Becton Dickinson)^16^. After vigorous vortexing to ensure sample homogenization, two drops were used to make a smear for Ziehl Neelsen staining, and the remainder divided equally for MGIT, MODS, and Xpert MTB/RIF. Samples for Xpert MTB/RIF analysis were promptly frozen at −80 °C for analysis at the end of the study.

Liquid mycobacterial culture was performed using the MGIT 960 system and MODS assay. A laboratory technician (RM) first received one month MODS training at the Universidad Peruana Cayetano Heredia in Lima, Peru. Standard protocols were followed^15,16^, except that MODS plates were examined 3 times a week rather than daily for logistic reasons.

Positive cultures from both methods were identified as *M*. *tuberculosis* complex (MTBC) or non-MTBC by the BD MGIT TBc Identification test (BD Diagnostics, Sparks, MD, USA). They were then further speciated and probed for isoniazid and rifampicin resistance mutations by PCR, using the Hain Genotype® line probe assay platform (Hain Lifescience GmbH, Nehren, Germany). The Xpert MTB/RIF assay (version G4) was performed at the end of the study on specimens from all children treated for confirmed, highly probable or possible TB, and from children in whom TB had been excluded. Laboratory procedures were externally monitored through the UK NEQAS quality assurance scheme and annual Good Clinical Laboratory Practice audits (Qualogy, UK).

In order to compare the performance of MGIT and MODS, and to prevent observer bias in the interpretation of either result, laboratory technologists were blinded to the identity of the MODS portion of each specimen. Specimens were instead identified by an electronically generated random numeric code, the key to which was held by the Principal Investigator.

### Statistical analysis

Data were analyzed at both the patient and the specimen level. We first performed a *per patient* analysis to compare the sensitivity of smear microscopy, MGIT, MODS and Xpert MTB/RIF for identification of culture confirmed pulmonary TB cases. We then included all specimens (pulmonary and extra-pulmonary) in a *per specimen* analysis to calculate the sensitivity of each method against the existing reference standard of a positive *M*. *tuberculosis* culture (by MGIT and/or MODS).

To explore the specificity of Xpert MTB/RIF we calculated the proportion of specimens from Not TB cases that were Xpert MTB/RIF positive. Having established the very high specificity of the Xpert MTB/RIF assay, we then repeated the *per specimen* analysis using a composite reference standard incorporating Xpert MTB/RIF, such that a specimen was considered positive if either culture or Xpert MTB/RIF identified *M*. *tuberculosis*.

We used McNemar’s χ^2^ test to compare proportions between tests on paired aliquots of the same sample. To investigate any learning effect as laboratory staff became more experienced with MODS we performed the χ^2^ test for trend in the proportion of *M*. *tuberculosis* culture positive specimens identified by MODS in each quarter, and the Wilcoxon rank sum test to compare MODS time to detection (TTD) in the first and second years of the study. We used the Wilcoxon signed rank test to compare TTD of MTBC by MODS and MGIT. Associations with a positive Xpert MTB/RIF assay were explored using univariable and multivariable logistic regression models adjusted for clustering at the patient level.

### Ethical approval and informed consent

The study was approved by the Kenya National Ethics Committee. All study procedures were performed in accordance with relevant guidelines and regulations. A parent or guardian provided written informed consent.

### Data availability

The datasets generated during and/or analysed during the current study are available from the corresponding author on reasonable request.

## Results

**Figure 2:**
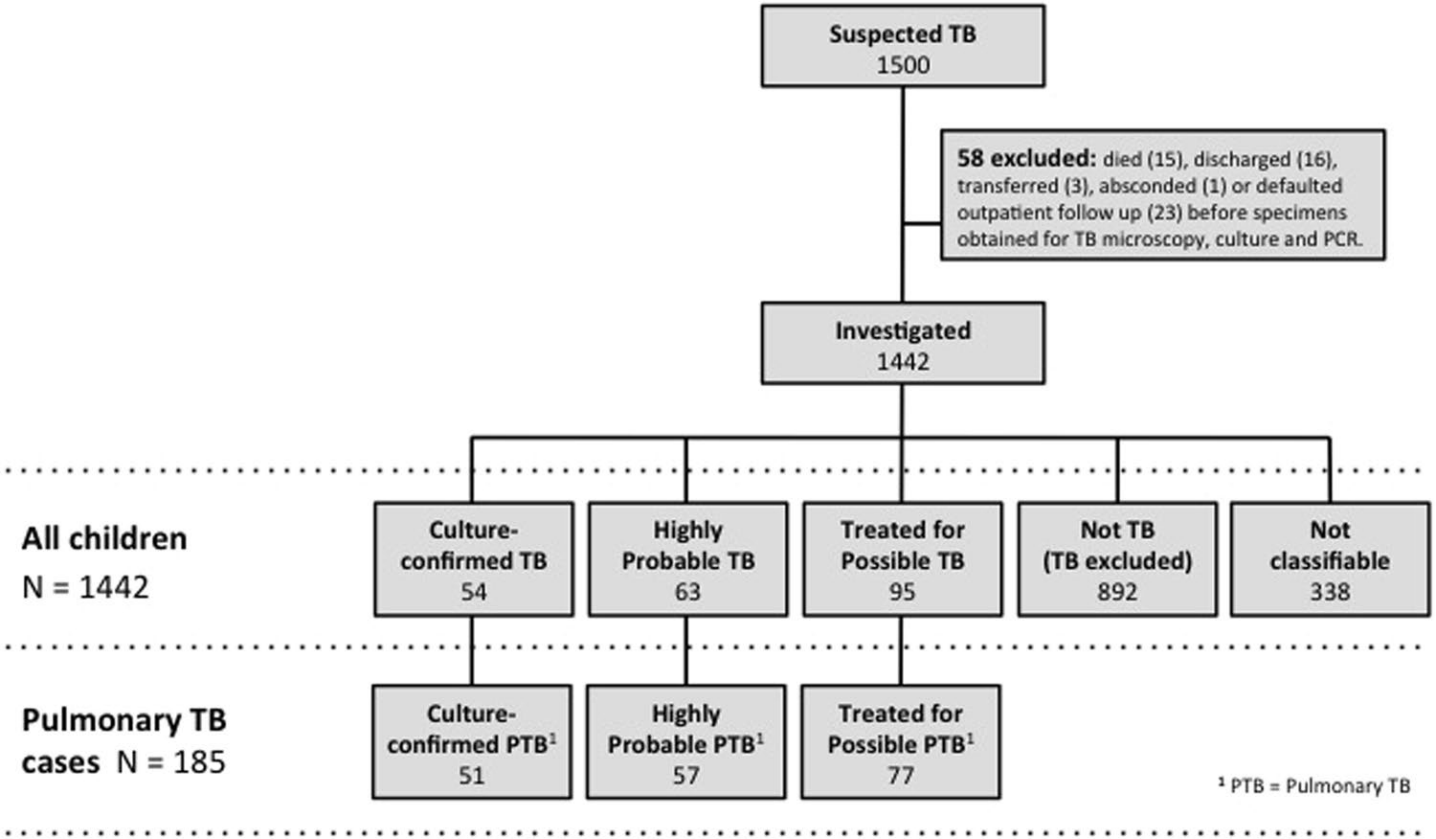
Patient enrolment and disease assignments.

We identified 1500 children with features of suspected TB, 1442 of whom were investigated for TB (Fig. 2). Active TB was diagnosed in 212 (14.7%) children, including 54 (25.5%) with Culture-confirmed TB, 63 (29.7%) with Highly Probable TB, and 95 (44.8%) with Possible TB. Most cases (185, 87%) had pulmonary TB (PTB). Baseline demographic and clinical characteristics are summarized in Table 1. In keeping with previous studies, the proportion of cases that were bacteriologically confirmed was higher among older children.

**Table 1:** Baseline characteristics of children with and without TB.

	Confirmed TB	Highly Probable TB	Treated for Possible TB	Not TB (TB excluded)	Not classifiable
	(n = 54)	(n = 63)	(n = 95)	(n = 892)	(n = 338)
***Patient demographics***					
**Age**					
Median (IQR^1^), months	53 (21–112)	32 (13–61)	17 (10–68)	16 (9–40)	17 (9–48)
0 to 4 years	27 (50%)	47 (75%)	67 (70%)	750 (84%)	271 (80%)
5 to 9 years	14 (26%)	10 (16%)	15 (16%)	95 (11%)	45 (13%)
10 to 14 years	13 (24%)	6 (9%)	13 (14%)	47 (5%)	22 (7%)
Male sex	33 (61%)	32 (51%)	48 (51%)	469 (53%)	188 (56%)
***Clinical features***					
Cough > 2 weeks	40 (74%)	37 (59%)	71 (75%)	425 (48%)	194 (57%)
Fever > 2 weeks	37 (69%)	24 (38%)	66 (69%)	375 (42%)	167 (49%)
Weight loss/FTT^2^ > 4 weeks	32 (59%)	29 (46%)	51 (54%)	385 (43%)	168 (50%)
Pneumonia not responding to first line antibiotics	22 (41%)	21 (33%)	35 (37%)	260 (29%)	144 (43%)
Close TB contact	24 (44%)	25 (40%)	23 (24%)	145 (16%)	62 (18%)
HIV infected	15 (28%)	15 (24%)	35 (37%)	121 (14%)	88 (26%)
Severely malnourished	19 (35%)	31 (49%)	40 (42%)	303 (34%)	119 (35%)
Pulmonary TB^3^	51 (94%)	57 (90%)	77 (81%)	—	—
Extra-pulmonary TB^3^	7 (13%)	6 (10%)	20 (21%)	—	—

^1^IQR, interquartile range.

^2^FTT, failure to thrive.

^3^Six patients with pulmonary TB also had extrapulmonary TB (4 with culture confirmed TB, 2 that were treated for possible TB).

### Per patient analysis of pulmonary TB cases

There were 51 cases of Culture-confirmed pulmonary TB. Smear microscopy, MGIT and MODS on the *initial* sputum specimen identified *M*. *tuberculosis* in 13 (25%), 40 (78%) and 33 (65%) cases, respectively (Table 2). Sensitivity was highest for each method among smear positive samples; Xpert MTB/RIF identified 100% of smear-positive cases and 53% of smear-negative cases on the first sputum specimen (Table 2).

**Table 2:** Sensitivity of each method for detection of *M*. *tuberculosis* in the initial sputum specimen from patients with culture confirmed pulmonary TB.

	AFB smear positive (N = 13)		AFB smear negative (N = 38)		Total (N = 51)	
	N	Sensitivity (95% CI)	N	Sensitivity (95% CI)	N	Sensitivity (95% CI)
AFB smear microscopy	13	N/A	N/A	N/A	13	25.5 (14.3–39.6)
MGIT culture	13	100.0 (75.3–100.0)	27	71.1 (54.1–84.6)	40	78.4 (64.7–88.7)
MODS culture	13	100.0 (75.3–100.0)	20	52.6 (35.8–69.0)	33	64.7 (50.1–77.6)
MGIT + MODS culture	13	100.0 (75.3–100.0)	30	78.9 (62.7–90.4)	43	84.3 (71.4–93.0)
Xpert MTB/RIF	13	100.0 (75.3–100.0)	20	52.6 (35.8–69.0)	33	64.7 (50.1–77.6)

Second and third sputum specimens were obtained from 237 (16%) and 102 (7%) children, respectively. Eight (16%) culture confirmed PTB cases were culture negative from the initial sample but grew *M*. *tuberculosis* from a subsequent sputum sample. MGIT, MODS and Xpert MTB/RIF each demonstrated a modest incremental yield from additional specimens (Table 3). None of the 8 *initial* culture-negative specimens from confirmed PTB cases were Xpert MTB/RIF positive. However Xpert MTB/RIF did detect *M*. *tuberculosis* in sputum from 5 additional children with culture negative pulmonary TB (3 with clinically Highly Probable TB and 2 who were treated for Possible TB).

**Table 3:** Cumulative sensitivity (incremental yield) of serial sputum specimens from patients with culture confirmed pulmonary TB.

Specimen	N	Smear microscopy		MGIT culture		MODS culture		MODS + MGIT culture		Xpert MTB/RIF	
		Test + (%)	Cum. yield*	Test + (%)	Cum. yield*	Test + (%)	Cum. yield*	Test + (%)	Cum. yield*	Test + (%)	Cum. yield*
1^st^ specimen	51	13 (25%)	25%	40 (78%)	78%	33 (65%)	65%	43 (84%)	84%	33 (65%)	65%
2^nd^ specimen	32	10 (31%)	33%	22 (69%)	82%	15 (68%)	73%	24 (75%)	90%	18 (56%)	69%
3^rd^ specimen	23	7 (30%)	33%	14 (61%)	84%	13 (57%)	75%	15 (65%)	92%	13 (57%)	71%

*Cum. yield = cumulative sensitivity of serial sputum specimens. In 4 (8%) cases of culture-confirmed pulmonary TB, culture confirmation was obtained from a subsequent specimen (n = 1) or from a specimen excluded from the analysis because MODS culture was not performed (n = 3).

### Per specimen analysis of pulmonary and extra-pulmonary specimens combined

A total of 2085 specimens were obtained for mycobacterial culture. We were unable to do MODS on 182 samples due to holiday staffing shortages (169) or insufficient sample (13). A total of 1903 specimens were therefore included in the analysis, of which 1802 (95%) were sputum specimens, 33 (2%) were smear positive, and 97 (5%) were *M*. *tuberculosis* culture positive (Supplementary Appendix, Table S1). MGIT was more sensitive than MODS overall (93.8% vs 76.3%, p = 0.002), among all sputum specimens, among induced sputum specimens, and among specimens from both younger and older children (Table 4).

**Table 4:** Comparison of MGIT and MODS culture sensitivity against culture reference standard incorporating both MGIT and MODS.

	MGIT positive		MGIT negative		MGIT Sensitivity % (95% CI)	MODS Sensitivity % (95% CI)	p value
	MODS positive	MODS negative	MODS positive	MODS negative			
**Specimen type**							
All specimens	68	23	6	1807	93.8 (87.0–97.7)	76.3 (66.6–84.3)	0.002
All sputum specimens	61	22	6	1713	93.3 (85.9–97.5)	75.3 (65.0–83.8)	0.003
Induced sputum	40	18	6	1603	90.6 (80.7–96.5)	71.9 (59.2–82.4)	0.014
**Age of patient**							
<5 years	28	14	5	1398	89.4 (76.9–96.5)	70.2 (55.1–82.7)	0.039
5–14 years	36	8	1	380	97.8 (88.2–99.9)	82.2 (67.9–92.0)	0.039

There was weak evidence of an increase in MODS sensitivity (χ^2^ test for trend p = 0.038) and a decrease in TTD over time, with a median TTD of 16 (IQR 7 to 21) days in 2010 compared with 10 (7 to 14) days in 2011 (p = 0.052; Supplementary Appendix, Fig. S1). Overall, TTD was slightly shorter for MODS than MGIT (11 [IQR 6 to 15] days versus 12 [7 to 17] days, p = 0.001).

Discordant culture results were obtained for 29 specimens. Independent bacteriological confirmation of *M*. *tuberculosis* was available for 21/29 of these specimens, either by the Xpert MTB/RIF assay on the original specimen (15/29) and/or by isolation of *M*. *tuberculosis* from an independent specimen from the same patient (Supplementary Appendix, Table S2). The clinical picture strongly supported the presence of *M*. *tuberculosis* complex in the remaining 8 culture-discordant samples: 5 MGIT+/MODS− specimens came from children with clinically Highly Probable TB prior to culture results; 2 MGIT−/MODS+ specimens came from children treated empirically for TB prior to the culture result although they did not meet the stringent definition of Highly Probable TB; and one MGIT+/MODS− specimen that grew *M*. *bovis* BCG came from a 4 month old HIV-infected child who had a clinical syndrome consistent with disseminated BCG disease (Supplementary Appendix, Table S2). All were the only positive cultures in their batch, arguing against cross-contamination causing the discordance. Even if cross-contamination were the cause and these specimens considered culture negative, MGIT would remain more sensitive than MODS (95.5% vs 80.9%, p = 0.005).

Xpert MTB/RIF identified 72/97*M*. *tuberculosis* culture positive specimens, giving a sensitivity compared to culture of 74.2% (95% CI 64.3 to 82.6). Xpert MTB/RIF was also positive on 5/86 (6%) specimens from patients with culture-negative but clinically highly probable TB, and 2/179 (1%) specimens from children treated for possible TB. No other specimens were Xpert MTB/RIF positive, including none of 1164 specimens obtained from children in whom TB was subsequently excluded - giving a specificity of 100% (1 sided 97.5% CI 99.7 to 100%) and strongly suggesting positive Xpert MTB/RIF results should be interpreted as true positives.

Against the composite reference standard of a positive TB culture and/or Xpert MTB/RIF result, the sensitivity of TB culture was 93.3% (86.6 to 97.3), compared with 76.0% (66.6 to 83.8) for Xpert MTB/RIF (p = 0.002; Table 5). The sensitivity of MGIT and MODS culture alone were 87.5% (79.6 to 93.2) and 71.2% (61.4 to 79.6), respectively.

**Table 5:** Comparison of culture and Xpert MTB/RIF sensitivity against composite reference standard of both culture and Xpert MTB/RIF.

	Culture positive		Culture negative		Culture Sensitivity % (95% CI)	Xpert MTB/RIF Sensitivity % (95% CI)	p value
	Xpert positive	Xpert negative	Xpert positive	Xpert negative			
**Specimen type**							
All specimens	72	25	7	1452	93.3 (86.6–97.3)	76.0 (66.6–83.8)	0.002
All sputum specimens	68	21	7	1419	92.7 (85.6–97.0)	78.1 (68.5–85.9)	0.008
Induced sputum	47	17	5	1356	92.8 (83.9–97.6)	75.4 (63.5–84.9)	0.011
**Age of patient**							
<5 years	32	15	7	1152	87.0 (75.1–94.6)	72.2 (58.4–83.5)	0.088
5–14 years	40	10	0	300	100 (92.9–100)	80.0 (66.3–90.0)	0.002

Table 6 shows Xpert MTB/RIF sensitivity against the composite reference standard, broken down by smear status, specimen type, age and HIV status. Sensitivity was higher for smear positive specimens (100% compared with 65% for smear negative specimens, p < 0.001), with a trend towards higher sensitivity among sputum samples. Neither age nor HIV status were associated with Xpert MTB/RIF yield in univariable or multivariable analyses.

**Table 6:** Sensitivity of the Xpert MTB/RIF assay against the composite reference standard.

	No. MTBC positive specimens	No. Xpert MTB/RIF positive	Xpert MTB/RIF Sensitivity, % (95% CI)		p value
**Specimen type**					
sputum specimens	96	75	78.1	(68.5 to 85.9)	0.062
non-sputum specimens	8	4	50.0	(15.7 to 84.3)	
**Specimen smear status**					
smear positive	33	33	100.0	(89.4 to 100.0)	<0.001
smear negative	71	46	64.8	(52.5 to 75.8)	
**Patient age**					
0–4 years	54	39	72.2	(58.4 to 83.5)	0.461
5–14 years	50	40	80.0	(66.3 to 90.0)	
**Patient HIV status**					
HIV positive	19	15	78.9	(54.4 to 93.9)	0.725
HIV negative	85	64	75.3	(64.7 to 84.0)	
**All MTBC positive specimens**	104	79	76.0	(66.6–83.8)	—

Xpert MTB/RIF identified rifampicin resistance in 4 specimens from the same child, all of which were confirmed resistant to rifampicin by MODS and the GenoType MTBDR*plus* assay. MODS did not identify any other rifampicin resistant isolates.

## Discussion

The lack of reliable diagnostic tools is the single greatest obstacle to improved childhood TB case management, particularly in low resource settings where the disease burden is greatest. Expansion of TB laboratory diagnostic capacity provides an opportunity to improve both diagnosis and surveillance in many settings^3^. However good quality data on the performance of each method are required to optimize their use and interpretation.

Few studies have compared laboratory methods for childhood TB diagnosis. Limitations of existing studies include small numbers of confirmed TB cases^5–7,14,17–22^, particularly among young children in whom diagnosis is most challenging^5–7,14,19,22^, and exclusion of ‘gold standard’ methods for specimen collection and/or mycobacterial culture^5–7,17,20,23–26^. Strengths of this study include the use of both sputum induction and liquid mycobacterial culture in keeping with current international recommendations^2^, and rigorous diagnostic assignments based on detailed clinical assessment and follow up. Prospective recruitment of all children who met broad, pre-defined inclusion criteria also ensures generalizability to a wide range of settings and clinical syndromes, including among children under 5 years who accounted for 81% of those investigated and half of all confirmed cases.

While comparison of culture yield between studies is hampered by widely varying clinical definitions, specimen types and culture methods, the proportion of culture-confirmed cases in our study is consistent with other studies using liquid mycobacterial culture for paediatric diagnosis^5,8,14,18,20,26–30^.

In our study MGIT was more sensitive than MODS culture. This is in keeping with the only other published study comparing MGIT with MODS for paediatric TB diagnosis^5^. Importantly we were also able to demonstrate the higher yield of MGIT for culture of paediatric induced sputum samples. These results contrast with other studies in adults in which MODS compared more favourably with MGIT^4^. The paucibacillary nature of childhood TB may have a role, and variation between centres also suggests MODS performance is operator dependent^4^. In support of this, even after one month residential training with an experienced team, we were able to demonstrate a learning effect with increasing MODS yield and reducing time to detection during the two years of the study. We did not compare MODS with culture on solid media, however other studies have shown MODS to be superior to Lowenstein Jensen media for diagnosis of childhood TB^6,7,26^.

Against a reference standard of mycobacterial culture, Xpert MTB/RIF sensitivity was similar to other paediatric studies^11^, and comparable to MODS - in keeping with the only other study to compare the two methods^29^. Significantly, a combination of culture and Xpert MTB/RIF increased bacteriological yield without compromising specificity.

Importantly, we were able to confirm the very high specificity of Xpert MTB/RIF in a large sample of specimens from children in whom TB had been excluded by careful clinical, radiological and laboratory assessment and follow up. Positive Xpert MTB/RIF results have been reported in a small number of child TB suspects who did well at follow up without TB treatment^11,24^. However the very high precision with which we were able to demonstrate 100% specificity of Xpert MTB/RIF in a carefully characterized group of children without TB suggests that these apparent false positive results from TB suspects in very high burden settings may have represented self-limiting pulmonary tuberculosis. This is well recognized in the pre-chemotherapy literature^31^.

Our primary analysis compared Xpert MTB/RIF sensitivity with the current culture reference standard. Having demonstrated the very high specificity of Xpert MTB/RIF we then repeated the analysis using a composite reference standard of positive culture or Xpert MTB/RIF. Composite reference standards should be used with care^32^. However, given the equivalent high specificities of Xpert MTB/RIF and mycobacterial culture, incorporation bias in favour of Xpert MTB/RIF is unlikely. Furthermore, misclassification bias arising from the imperfect sensitivity of culture may bias estimates of the accuracy of Xpert MTB/RIF culture alone is used as the reference^33,34^. We believe the composite reference standard therefore provides a fairer comparison of the two methods, and a better estimate of the true sensitivity of the Xpert MTB/RIF assay.

One potential limitation of our study is that molecular typing was not performed on MGIT-MODS discordant culture isolates to exclude cross-contamination. Nevertheless there was strong microbiological and/or clinical evidence to corroborate the positive culture in every case. In addition, negative controls in every MGIT batch and MODS plate provided no evidence of cross-contamination throughout the study, consistent with the very low prevalence and concentrations of *M*. *tuberculosis* in these paediatric specimens.

Due to finite resources, most children only had a single sputum sample obtained. Although this and other studies show the incremental yield of additional specimens where obtained, analysis of a single specimen is likely to reflect practice in most low resource settings due to the incremental cost of processing additional specimens.

In conclusion, our results underline the imperfect sensitivity of all currently available methods for bacteriological diagnosis of childhood TB. The choice of method in any particular setting depends on several factors including prevalence of TB and drug resistance; resources available to risk stratify patients for testing; test sensitivity, cost^35^, time to detection; and the availability of trained staff and laboratory biosafety facilities. Our data confirm the superior sensitivity of MGIT compared with MODS and Xpert MTB/RIF. Although MODS may have a role in some settings, the operationally simpler Xpert MTB/RIF assay was as sensitive as MODS, demonstrated equivalent specificity to MGIT, and combined with MGIT it optimized bacteriological yield. Together these results strengthen the evidence base for inclusion of Xpert MTB/RIF in the reference standard for bacteriologically confirmed childhood TB in both WHO clinical guidelines^35^ and research.

### Role of the funding source

The study was supported by the Wellcome Trust in the form of research fellowships to AJB (081697) and JAGS (098532), and a core grant to the KEMRI-Wellcome Trust research programme (077092); and by a project grant from the Pneumonia Etiology Research for Child Health (PERCH) project funded by the Bill & Melinda Gates Foundation at Johns Hopkins Bloomberg School of Public Health. The funders had no role in data analysis or in the decision to publish.

## Electronic supplementary material

Supplementary Appendix
