# Author Correction: Bacteriological diagnosis of childhood TB: a prospective observational study

**DOI:** 10.1038/s41598-018-25250-w

**Published:** 2018-05-03

**Authors:** Andrew J. Brent, Daisy Mugo, Robert Musyimi, Agnes Mutiso, Susan C. Morpeth, Michael Levin, J. Anthony G. Scott

**Affiliations:** 1 KEMRI-Wellcome Trust Research Programme, Kilifi, Kenya; 2 University of Oxford, Oxford, UK; 3 Imperial College London, London, UK; 4 Oxford University Hospitals NHS Foundation Trust, Oxford, UK; 5 London School of Hygiene & Tropical Medicine, London, UK

Correction to: *Scientific Reports* 10.1038/s41598-017-11969-5, published online 18 September 2017

The original version of this article contained an error in the spelling of the author Susan C Morpeth, which was incorrectly given as Susan Morpeth.

Additionally, in this Article, an affiliation for Susan C Morpeth was omitted. The correct affiliations for Susan C Morpeth are listed below:

KEMRI-Wellcome Trust Research Programme, Kilifi, Kenya

London School of Hygiene & Tropical Medicine, London, UK

These errors have now been corrected in the PDF and HTML versions of the paper, and in the accompanying Supplementary Information.

