# Supplementary material for: Bacteriological diagnosis of childhood TB: a prospective observational study
Source: Sci Rep. 2017 Sep 18;7:11808. doi: 10.1038/s41598-017-11969-5 (PMC5603584; doi:10.1038/s41598-017-11969-5)
Supplement: Supplementary file 1 — Supplementary Appendix [file 41598_2017_11969_MOESM1_ESM.doc]

**Bacteriological diagnosis of childhood TB: a prospective observational study**

Andrew J. Brent, Daisy Mugo, Robert Musyimi, Agnes Mutiso, Susan C Morpeth, Michael Levin, J. Anthony G. Scott

**Supplementary Appendix**

Table S - . *M. tuberculosis* yield of MGIT culture, MODS culture and the Xpert MTB/RIF assay among all specimens by specimen type and AFB smear result.

| **Specimen type** | **No. specimens** | | | **AFB smear positive (n=33)** | | | | | | **AFB smear negative (n=1870)** | | | | | | **Total** | | | | | |
| --- | --- | --- | --- | --- | --- | --- | --- | --- | --- | --- | --- | --- | --- | --- | --- | --- | --- | --- | --- | --- | --- |
|  | **Total** | **Smear positive** | | **MGIT culture** | | **MODS culture** | | **Xpert MTB/RIF*** | | **MGIT culture** | | **MODS culture** | | **Xpert MTB/RIF*** | | **MGIT culture** | | **MODS culture** | | **Xpert MTB/RIF*** | |
| ***Sputum specimens*** |  |  |  |  |  |  |  |  |  |  |  |  |  |  |  |  |  |  |  |  |  |
| expectorated | 135 | 15 | (11%) | 15 | (100%) | 14 | (93%) | 15 | (100%) | 10 | (8%) | 7 | (6%) | 8 | (7%) | 25 | (19%) | 21 | (16%) | 23 | (17%) |
| induced | 1667 | 16 | (1%) | 16 | (100%) | 14 | (88%) | 16 | (100%) | 42 | (3%) | 32 | (2%) | 36 | (2%) | 58 | (3%) | 46 | (3%) | 52 | (3%) |
| ***Other specimens*** |  |  |  |  |  |  |  |  |  |  |  |  |  |  |  |  |  |  |  |  |  |
| abscess fluid/pus | 22 | 1 | (5%) | 1 | (100%) | 0 | (0%) | 1 | (100%) | 1 | (5%) | 1 | (5%) | 0 | (0%) | 1 | (9%) | 1 | (4%) | 1 | (9%) |
| ascitic fluid | 13 | 0 | (0%) |  | N/A |  | N/A |  | N/A | 1 | (8%) | 1 | (8%) | 0 | (0%) | 1 | (8%) | 1 | (8%) | 0 | (0%) |
| CSF | 19 | 0 | (0%) |  | N/A |  | N/A |  | N/A | 0 | (0%) | 0 | (0%) | 0 | (0%) | 0 | (0%) | 0 | (0%) | 0 | (0%) |
| gastric aspirate | 3 | 0 | (0%) |  | N/A |  | N/A |  | N/A | 0 | (0%) | 0 | (0%) | 0 | (0%) | 0 | (0%) | 0 | (0%) | 0 | (0%) |
| joint aspirate | 1 | 0 | (0%) |  | N/A |  | N/A |  | N/A | 0 | (0%) | 0 | (0%) | 0 | (0%) | 0 | (0%) | 0 | (0%) | 0 | (0%) |
| lymph node FNA | 2 | 0 | (0%) |  | N/A |  | N/A |  | N/A | 0 | (0%) | 0 | (0%) | 0 | (0%) | 0 | (0%) | 0 | (0%) | 0 | (0%) |
| lymph node biopsy | 4 | 0 | (0%) |  | N/A |  | N/A |  | N/A | 0 | (0%) | 0 | (0%) | 0 | (0%) | 0 | (0%) | 0 | (0%) | 0 | (0%) |
| pericardial fluid | 3 | 0 | (0%) |  | N/A |  | N/A |  | N/A | 1 | (33%) | 1 | (33%) | 0 | (0%) | 1 | (33%) | 1 | (33%) | 0 | (0%) |
| pleural fluid | 21 | 1 | (4%) | 1 | (100%) | 1 | (100%) | 1 | (100%) | 2 | (9%) | 2 | (9%) | 2 | (10%) | 3 | (14%) | 3 | (14%) | 3 | (14%) |
| tissue biopsy | 2 | 0 | (0%) |  | N/A |  | N/A |  | N/A | 0 | (0%) | 0 | (0%) | 0 | (0%) | 0 | (0%) | 0 | (0%) | 0 | (0%) |
| urine | 11 | 0 | (0%) |  | N/A |  | N/A |  | N/A | 1 | (9%) | 1 | (9%) | 0 | (0%) | 1 | (9%) | 1 | (9%) | 0 | (0%) |
| Total | 1903 | 33 | (2%) | 33 | (97%) | 29 | (85%) | 33 | (100%) | 58 | (3%) | 45 | (2%) | 46 | (2%) | 90 | (5%) | 74 | (4%) | 79 | (4%) |

*Xpert MTB/RIF was performed on all specimens from children treated for confirmed, highly probable or possible TB, and on 1164 specimens from children without TB.

Table S - . Clinical and microbiological profiles of MODS-MGIT discordant cultures.

| **Results from discordant specimen** | | | **Independent specimen from same patient positive for *M. tuberculosis*** | **Diagnosis based on all other available clinical and microbiological data (excluding discordant culture)** | **No. patients (total 29)** |
| --- | --- | --- | --- | --- | --- |
| **MGIT culture** | **MODS culture** | **Xpert MTB/RIF** |
| Positive | Negative | Positive | Yes | Confirmed TB | 7 |
| Positive | Negative | Positive | No | Confirmed TB | 5 |
| Positive | Negative | Negative | Yes | Confirmed TB | 5 |
| Positive | Negative | Negative | No | Highly Probable TB | 4 |
| Positive | Negative | Error | No | 1 Highly Probable TB, 1 Disseminated BCG | 2 |
| Negative | Positive | Positive | Yes | Confirmed TB | 3 |
| Negative | Positive | Positive | No | - | 0 |
| Negative | Positive | Negative | Yes | Confirmed TB | 1 |
| Negative | Positive | Negative | No | Possible TB (treated) | 2 |

Figure S - . MODS learning effect: Proportion of *M. tuberculosis* complex (MTBC) positive cultures identified by MODS and MODS time to detection by quarter.
